# Defining fatigue from the experiences of patients living with chronic fatigue

**DOI:** 10.3389/fmed.2024.1429275

**Published:** 2024-08-12

**Authors:** Carielle Joy Rio, Gina M. Gehling, Catherine Blumhorst, Alexander Ross, Leorey N. Saligan

**Affiliations:** ^1^Division of Intramural Research, National Institute of Nursing Research, National Institutes of Health, Bethesda, MD, United States; ^2^College of Nursing, University of Florida, Gainesville, FL, United States

**Keywords:** chronic fatigue, symptom experience, defining fatigue, chronic symptoms, patient reported symptoms

## Abstract

**Introduction:**

Fatigue is a multidimensional, highly individualized symptom experience perceived by people, regardless of health status. It is the most common complaint among those seeking primary care, yet, despite being a frequently reported symptom, it remains poorly understood.

**Methods:**

This is an exploratory study utilizing a qualitative descriptive approach that aims to explore the description of fatigue from the personal experiences of 16 participants living with chronic fatigue. Themes were generated from transcripts of in-depth interviews that focused on a central question: “how would you describe your fatigue from your own experience?”

**Results:**

Analysis of the participants’ interview transcripts revealed three themes. The first theme focused on fatigue as a unique personal experience, which included experiential descriptions or measures of fatigue that the participants used to describe their symptoms. The second theme focused on fatigue as an experience beyond self, which highlighted the consequences of fatigue on interpersonal interactions and the performance of social roles, as well as the potential of utilizing social support to cope with the limitations caused by this condition. The last theme was on living with fatigue, which focused on ways participants attempted to discern their condition and manage the consequences of fatigue.

**Discussion:**

Experiences of chronic fatigue have patterns and personal meanings that vary between individuals. Caring for persons experiencing chronic fatigue requires acknowledgment of unique personal experiences and coping strategies. Due to the nature of the method, the results of this study are not generalizable and only reflect the experiences of the participants.

## 1 Introduction

Fatigue is a multidimensional, highly individualized experience perceived by both healthy people and those experiencing illness that varies in duration and severity (1–3). It is defined as a state of exhaustion caused by a reduction in physical and/or mental capability that causes unrelenting and pervasive tiredness or weakness that is not relieved by rest that negatively impacts a person’s ability to manage typical daily activities (1, 4). Fatigue is often described as an overwhelming and debilitating experience that may be sustained or fluctuating (5, 6). The physiologic mechanism of fatigue has been closely linked with inflammation which induces alteration in the activation of neuronal processes. Changes in the mesolimbic reward system have also been linked with fatigue related to a decrease in or lack of motivation (7).

Globally, it is estimated that 20% of adults experience general fatigue (less than 6 months in duration or for an unspecified period), while 10% experience chronic fatigue (8). While many people have experienced what they report to be “fatigue” on occasion, this contrasts with what constitutes as “being tired” due to the latter’s transient nature and relief with rest. Currently, no standard definition of fatigue exists (4). Fatigue is the most common complaint among those seeking primary care and has been associated with both acute and chronic conditions (i.e., common cold, influenza, cancer, fibromyalgia, rheumatoid arthritis, systemic lupus erythematosus, Parkinson’s disease, and multiple sclerosis) (9–17). Studies show that fatigue affects 50% of individuals with heart failure (18), 72% of those experiencing long COVID (19), and up to 98% of those diagnosed with myeloma (20). Reports of fatigue are also often captured in tandem with other symptomology associated with acute and chronic illnesses (5).

Chronic fatigue, specifically, is defined as fatigue that persists for at least 6 months which may be present with or without any known cause (21). It is important to distinguish chronic fatigue from myalgic encephalomyelitis/chronic fatigue syndrome (ME/CFS). The case definition of ME/CFS was first set by the US Centers for Disease Control and Prevention (CDC) in 1994. Using the Fukuda Criteria, the CDC defined fatigue as having at least four of the symptoms (impaired short-term memory or concentration, sore throat, lymphadenopathy, myalgia, multiple joint pains without swelling, headache presenting as a new type, pattern or severity, unrefreshing sleep, and post-exertional fatigue or malaise lasting at least 24 h) for 6 months or more (22). While the earliest definition of ME/CFS was primarily focused on symptoms, a more recent criteria introduced by the National Academy of Medicine (NAM) provided a more holistic concept of ME/CFS. NAM 2015 clinical diagnostic criteria for ME/CFS include “substantial reduction/impairment in the ability to engage in pre-illness levels of occupational, educational, social, or personal activities.” (23) However, these advances in establishing the diagnostic criteria are more focused on ME/CFS as a medical diagnosis but not on chronic fatigue as a symptom which is experienced by non-ME/CFS patients.

Despite being a frequently reported symptom, fatigue remains poorly understood, which makes it difficult to diagnose and treat (4, 24). Elucidating the prevalence and underlying biological etiology of fatigue, assessment, and treatment of fatigue remains difficult. It is important to understand and address fatigue as it poses a negative impact on family and other social relationships, as well as an individual’s ability to function and be productive (21, 25). Thus, this study explored the descriptions of fatigue from the experiences of adults who have been diagnosed and are living with chronic fatigue co-occurring with different medical conditions. This study aimed to shed light on how individuals with chronic fatigue describe their symptom experience. The identification of key themes and verbal descriptors may help guide the development of targeted techniques that assess and manage fatigue to improve patient outcomes and quality of life.

## 2 Materials and methods

### 2.1 Ethics statement

This study was the exploratory part of a quantitative observational study that recruited participants who were experiencing chronic fatigue (NCT03952624). The study was approved by the Institutional Review Board of the National Institutes of Health in Bethesda, Maryland, United States. Informed consent was obtained prior to conducting any of the interviews.

### 2.2 Procedures

To provide a deeper understanding of chronic fatigue from the perspective of the person experiencing the symptom, in-depth semi-structured interviews were conducted. The interviews focused on the central question “How would you describe the chronic fatigue you have experienced or are experiencing?” Aside from the general description, the participants were also asked to select from and expand upon *a priori* set of chronic fatigue descriptors obtained from published literature: unrelenting exhaustion, physical impairment, cognitive impairment, not related to physical activity, not relieved by rest, and interferes with usual functioning (26–28).

The interviews were conducted in English in a quiet room of the hospital’s outpatient unit with only the participant, the interviewer, and a research nurse in the room. To maintain consistency, the participants were all interviewed by one clinician trained by a licensed psychologist to conduct this specific interview. During the interviews, the interviewer presented the fatigue descriptors in the same format, in a consistent order where the fatigue descriptors were numbered to always place them in the same sequence. The participants were informed that the interviews were audio-recorded and then transcribed for analysis. After each interview, the audio files and transcriptions were transferred from our clinic computers to a secure directory with restricted access.

### 2.3 Sample

Purposive sampling was used, and individuals who have experienced chronic fatigue, defined as fatigue lasting for at least 6 months, were included in this study. The experience of chronic fatigue was the phenomenon of interest. The commonality among participants was their experience of chronic fatigue, although there were variations in their clinical and sociodemographic characteristics. There was no pre-determined number of samples for the exploratory qualitative component of the study. The participants’ narratives were analyzed after each interview, and additional participants were interviewed until saturation was noted. No new themes emerged during the analysis of the transcripts from the interview with the 14th participant. To ensure that saturation was achieved on the 14th participant, two additional participants were included in the analysis. Similarly, no new themes emerged during the analysis of the 15th and 16th participants; thus, a sample size of 16 was deemed sufficient. This exploratory analysis involved 16 participants with different medical diagnoses including six with cancer, four with SLE, two with neurodegenerative disease, three with ME/CFS, and one with fatigue of unknown origin.

### 2.4 Analysis

A qualitative descriptive approach was used to determine how individuals living with chronic fatigue described the condition based on their experiences. Unlike other qualitative research methods, the qualitative descriptive approach focuses on describing the “who, what, and where” an event or the participants’ experience of the phenomenon of interest (29). Other significant statements and themes that arose from the interviews aside from the descriptions of fatigue were included in the results. NVivo 13 (www.lumivero.com) was used to manage codes and themes during analysis. To ensure rigor and minimize the risk of bias, triangulation across analysts was done. The interview transcripts were independently analyzed by two researchers, and the results were reviewed by one research nurse and one nurse practitioner who have followed the participants throughout their participation in the parent study. No conflicts in the themes identified arose; however, meetings were conducted to reach a consensus as to how the themes were labeled. All team members involved in the interview and management/analyses of interview transcripts were trained by a credentialed clinical psychologist experienced in conducting interviews and managing/analyzing interview transcripts (Table 1).

**Table 1:** Characteristics of participants.

Participant code	Medical diagnosis	Highest education	Employment status	Income
01–98-001	Cancer	Some college, no degree	Self-employed, part-time/ underemployed	Refused
01–98-002	Cancer	Graduate / Professional	Employed Full-time	$100,000+
01–98-003	Cancer	Graduate / Professional	Employed Full-time	$100,000+
01–98-004	Cancer	Graduate / Professional	Self-employed, part-time/ underemployed	$15,000-24,999
01–98-006	Cancer	Graduate / Professional	Employed Part-time	Refused
01–98-007	Cancer	Graduate / Professional	Retired	$100,000+
03–98-002	ME/CFS	Graduate / Professional	Unemployed	$25,000 to $34,999
03–98-003	ME/CFS	Bachelor’s degree	Employed Part-time	Under $15,000
03–98-004	ME/CFS	Graduate / Professional	Employed Full-time	$100,000+
07–98-001	SLE	Bachelor’s degree	Employed Full-time	$50,000 to $74,999
07–98-002	SLE	Bachelor’s degree	Retired (medical disability)	$100,000+
07–98-003	SLE	Bachelor’s degree	Employed Full-time	$100,000+
07–98-004	SLE	Graduate / Professional	Employed Full-time	$100,000+
08–98-001	Neurodegenerative disease	Graduate / Professional	Retired	$50,000 to $74,999
08–98-002	Neurodegenerative disease	Graduate / Professional	Employed Part-time	$75,000 to $99,999
09–98-004	Fatigue of unknown origin	Graduate / Professional	Employed Full-time	$100,000+

## 3 Results

Three themes elucidate how individuals with chronic fatigue describe their fatigue experiences: (1) fatigue as a unique personal experience, (2) fatigue as an experience beyond self, and (3) living with chronic fatigue. These themes were drawn from the narratives of 16 individuals living with chronic fatigue. Of the 16 participants, 6 were diagnosed with cancer, 3 with myalgic encephalitis/chronic fatigue syndrome, 4 with systemic lupus erythematosus, 2 with neurodegenerative disease, and 1 with fatigue of unknown origin. The majority of the participants have completed post-baccalaureate education (master’s or doctoral degree) and were employed full-time at the time of study participation. The analysis of the narratives focused on how the participants’ subjective descriptions of fatigue regardless of their clinical and sociodemographic characteristics.

### 3.1 Theme 1: Fatigue as a unique personal experience

The participants have unique ways of describing their fatigue experience. Even when presented with *a priori* descriptors, they provided rich descriptions of fatigue based on experiences. We observed that descriptors reflecting various dimensions of fatigue and patterns of fatigue were highly individualized (Table 2). Interestingly, one participant remarked that asking patients to rate their exhaustion using a numeric scale may not be the best way to assess fatigue because exhaustion does not capture fatigue’s complexity and obstinate nature.

**Table 2:** Points to consider in planning care with persons experiencing chronic fatigue based on the participants’ experience.

Descriptors of fatigue	Drained Foggy Heaviness Worn out Hitting a wall Not feeling “right” Difficulty carrying out voluntary movements Having a “depleted” baseline level of energy
Ways to measure fatigue	How do you feel after waking up from a night of adequate sleep? How many times do you feel the need to nap during the day? How long do you need to nap to feel better? At what time during the day do you feel “worn out”? What activities are/were you able to do? How long can you do these activities before you start to feel you have hit a wall or cannot go on?
Ways to manage fatigue	Get enough sleep and take naps during the day Planning and pacing activities while allowing flexibility Using technology to aid activities of daily living Seek support to perform chores when needed Keeping a list of important things to remember

*“Exhaustion is something that is … it's something that you [experience due to a] cause and it disappears over time, but fatigue is something I don't believe disappears. It's something that stays there and that's what it feels like. It feels like you've run up against the wall and it doesn't matter what you do, that wall is still there so you can't get around it. You can't get through it. You can't get over it. You can't get under it. You just … you [are] just stuck with it.” (08-98-001)*

#### 3.1.1 Physical fatigue

From a physical perspective, some participants described fatigue as “feeling heavy.” Participant 01–98-007 expressed “*I feel like I’ve got weights on my body and then increase the gravitational constant.*” Similarly, another participant (08–98-001) also described their fatigue in reference to gravity “*It’s not a fact of being tired. It’s being like you got a weight on you so it’s like the gravity level has gone from 1 to 1 times gravity to 5 or 6 maybe 10 times gravity.*”

When asked what their fatigue felt like, participants described their fatigue experience as similar to the exhaustion one feels from flu that lingers for a while.

*“… when you have the flu and like even after you get better, but you're still wiped out for another week or so. That's what I um … that's what I experience.” (07-98-002).*

*“I'll get like body aches or like just the sort of feeling that you would feel when you're on the verge of a cold or whatever, and sometimes it's hard to tell like is that my immune system flaring up or am I starting to get a cold, um, because occasionally it will manifest that way.” (03-98-004)*

The resemblance of chronic fatigue symptoms with other conditions, such as the flu, is important to note as this may prevent clinicians from missing signs of chronic fatigue exacerbation when patients provide descriptions that overlap with other conditions.

The participants described fatigue as a general lack of energy and the inability to feel refreshed after a period of rest. *“You wake up tired. You go to bed tired. And you are just tired all day long, no matter what you do.”* (08–98-002). The link between physical activity and fatigue varies between participants. Some participants expressed that performing physical activity caused exacerbation of their fatigue, but some participants also noted that they constantly feel fatigued even without engaging in physical activity.

Two participants described fatigue as feeling chronically depleted and with a baseline energy level that is lower than those who are not experiencing fatigue. The statements below are exemplars of how individuals experiencing fatigue described their baseline energy levels.

*“Some of those, who when they wake up in the morning, [feel like] they have like 100% charge in their battery. I wake up, let's say at like 50% charged, and then throughout the day it's like everything [in] that battery goes down and even when I rest it doesn't always bring it back; like it doesn't always come back to 50% and I never get above 50%. So, it's just like you're always running at like sort of half capacity or less.” (03-98-003)*

*“I mean I just think of kind of like a battery, right? So, say if you have a good night's rest you should ideally be waking up like 80-90% or maybe even 100% charged. I feel like that can vary for me; maybe it's like 60 or 70[%] or so when I have a good night's rest. So, [with] my 60 to 70[%] I'm gonna tap out by the middle of the day or early afternoon every time because my energy level started so much lower.” (07-98-004)*

The battery analogy describes fatigue both in terms of energy reserve during the day and in terms of baseline energy level after what they considered to be ample periods of rest or sleep. Other general descriptions of chronic fatigue used during the interviews included feeling drained. *“I’m just drained. It’s just like, sigh, I cannot stay out, I cannot function like I… I cannot function. I cannot think about this I cannot think about what you are talking about, I cannot think about the TV, I just need to lay down and sleep.”* (01–98-001); and wiped out or worn out: *“There’s just like lack of that feeling of invigoration that I miss so greatly because if I go and walk, I do not feel invigorated. I feel deflated and wiped out and I cannot go for very long.”* (03–98-002). Fatigue was also compared to “hitting a wall”:

*“I think exhaustion is the is the wrong word because it [exhaustion] to me says that you actively are doing something to cause it. Fatigue, on the other hand, I think is something that is … It's like a wall that's there and it doesn't matter what you do the wall is still there it doesn't change it doesn't stop being there and there's nothing you can do to make it go away.” (08-98-001)*

#### 3.1.2 Cognitive fatigue

The participants also described their fatigue in terms of changes in cognitive function. Fatigue caused several participants to experience what they would describe as being “foggy.” According to some participants, cognitive changes during a fatigue episode often include losing the ability to follow complex texts and express one’s thoughts.

*“I can read something and it's like I can read all the words. All the words go in, but the meaning of it when I… by the time I get to the bottom of the page I can't connect the bottom of the page to the top of the page.” (03-98-002)*

One participant describes this experience as “*… feeling smart inside but having a curtain that fogs my brain from the world. It is frustrating to have big ideas but feeling unable to access these ideas”* (03–98-002). Another participant (01–98-004) described cognitive fatigue not only in terms of cognitive function but also in terms of *“Not being able to get my body to carry that out or there being a delay in what I’m thinking and being able to translate that into motion or movement.”*

Although 11 of the 16 participants are employed full-time and 2 are employed part-time, the cognitive changes often lead to limitations in social interactions because they may feel exhausted in carrying out conversations and have increased incidence of missing out on details that often affect their performance at work. The cognitive changes caused by fatigue not only affect people’s ability to perform complex tasks but can also impact their sense of identity. Participant 01–98-004 stated *“I used to feel like an intelligent human being, a competent person able to express my thoughts that I do not anymore since the past 4 years.”*

#### 3.1.3 Diurnal variability

We have also noted that the participants often used sleep as a benchmark to assess their own fatigue. For example, they described fatigue as feeling tired upon waking up in the morning despite having enough sleep and feeling unable to restart the following day. Fatigue means that one does not experience restoration of energy after a period of rest. Thus, some participants observed the number of times they would need to take a nap during the day. A higher number of daytime naps were associated with worse fatigue.

Analysis of the participants’ responses revealed that the severity of fatigue may vary throughout the day. However, we have not noted a single definitive pattern of fatigue occurrence or exacerbation across all participants or participants with similar medical diagnoses. Some participants experience the worst fatigue in the morning upon waking up, whereas other participants expressed that their fatigue peaks at noon through mid-afternoon and may continue to worsen in the evening.

### 3.2 Theme 2: Fatigue is an experience beyond self

#### 3.2.1 Impact of fatigue on interpersonal interactions and social roles

The impact of chronic fatigue on a person’s social wellbeing and the importance of social support were also highlighted by the participants (Table 2). Chronic fatigue causes a decrease in social interaction and family involvement. Physical and cognitive exhaustion may lead to difficulty in carrying out conversations with others. For example, one participant narrated:

*“I feel like it's very difficult to both express my thoughts of like how I think and feel and make connections with other people about what they're thinking because I can't access words and concepts and put things together that I've previously been able to do … and I can't remember simple little stupid words so sometimes that manifests just in terms of how I can't communicate with other people”* (01-98-004).

*“A lot of my friends and family complain that I hide out, they haven't heard from me in a while … I've had to apologize to friends and family quite a bit. I let them know I'm like this is just not a good time I'm tired or whatever.” (07-98-004)*

Participants also shared about how their condition affected their work performance as fatigue caused tardiness, missing task-related details, or being unable to complete tasks on time.

*“When I was typing on the computer, I just get exhausted, and I would say even though it was a simple thing I'm just hitting one key to the input data. It was difficult for me to do that because I was just, I just felt tired like my arms weighed tons.” (08-98-001)*

*“And I was sleeping between patients, couldn’t get my notes done.”* (07-98-004)

Although participants mentioned that fatigue affects their work performance, only one of the 16 participants discussed the financial impact of fatigue. In addition, people living with chronic fatigue had the tendency to push themselves to perform activities despite feeling unwell to meet the demands of social roles.

*“I can't … can no longer function in terms of trying to do any kind of household, work, trying to like to organize anything, try to do any kind of paperwork even pay a bill. I have to lay down.” (07-98-002)*

Some participants also found that explaining their condition and the limitations caused by fatigue was challenging. *“I just I cannot help but think about when I tell somebody that I’m really tired and they say, ‘I’m tired too’ … but it’s not the same tired.”* (08–98-002). They often feel misunderstood by people around them. It is vital to acknowledge the experiences of people who are living with fatigue. One of the challenges during interpersonal interactions shared by a participant is how fatigue is often labeled as an unimportant symptom.

*“There tends to be a mindset that's thrown our way that says like well I mean but you're not dead right like we saved your life but did you though like because how much of my life do I have access to how much of my sense of self do I have access to now it's a significantly limited portion of while yes I'm absolutely grateful that cancer didn't take me out I'm also incredibly pissed off that these quote ‘lifesaving drugs’ that were given to me took so much of my life away from me.” (01-98-004)*

As chronic fatigue affects not only the person experiencing it but also their families, colleagues, and wider social sphere, it is paramount that challenges to their emotional, social, and spiritual wellbeing are included in the assessment and care planning.

#### 3.2.2 The importance of social support

Social support is a vital resource for people as they adapt to limitations caused by chronic fatigue. Participants expressed that social support included practical help, such as spouses taking on more household roles, and neighbors helping with daily living activities like food preparation.

*“He (husband) has to pick up a lot of slack with stuff. I was having a flare, so he had to take over taking my son to school, picking him up because it was just too much for me to try to do it.” (07-98-002)*

They also shared how their support system prevents overexertion by providing reminders on activity limitations.

*“Sometimes I'll tell people like: ‘Okay, keep me in check’ because it's so easy to overdo and there's that delayed reaction, sometimes it would be immediate like you know but a lot of times it's an hour or 2 later or the next morning.” (03-98-004)*

Another form of social support is having family and/or friends listen to their experiences and allowing them to reflect on their progress. One participant recounted how honest observations expressed by a friend helped her track her symptoms.

*“The person who I shared the most of my experience with during my cancer journey … she was one of the few people who just kind of stuck around regularly during that time … she said, ‘Yeah I absolutely noticed there was a place during your cancer treatment where you dropped off.’ She said, ‘I just noticed you start losing your words and your flow wasn't there for you anymore.’ I hadn't had anybody say that to me.” (01-98-004)*

The participants’ narratives suggest that chronic fatigue impacts a person holistically. Chronic fatigue causes changes in the ability to start and/or complete activities which greatly impacts the participants’ performance at work, social functions, and hobbies. Cognitive changes such as feeling foggy and the inability to process textual information affect social function and performance of daily activities. The participants recognized that chronic fatigue impacts not only themselves but also their families, friends, and colleagues. Social support plays a role in helping the participants maintain their social roles and responsibilities and provides reminders regarding activity limitations to prevent exacerbation of their condition. However, due to the nature of chronic fatigue, wherein symptoms are variable and often present in manner that is difficult to describe, some participants expressed difficulty receiving recognition of their condition by others.

### 3.3 Theme 3: Living with chronic fatigue

One of the participants expressed never having been able to describe the fatigue experience despite having dealt with the symptoms for many years, thus describing her experience as a “kaleidoscope of horrors” due to its lack of predictable patterns or symptoms.

*“It feels like my body isn't coalesced properly; like I feel as if my physical energy is sort of leaking out everywhere … It's like if you've tried to fill a bucket with water but there are holes in it … My body feels like it can't hold the water in order to be able to use it as a tool. I feel like I can't hold my energy in order to be able to put it into place.” (01-98-004)*

Over time, the participants have learned to understand their condition, with its unique presentation and precipitating factors. They have also learned to recognize signs of exacerbation and ways to prevent exacerbation of their fatigue.

*“It's sort of like … I equated to you know people that get in debt. It's like once you're in debt it's easier to go further and spiral. With post exertional malaise, your limits shrink and then it becomes easy to overdo it further and get further in the hole. So, like having that headache [as one of the signs of exhaustion] is one of those things [that tell me] ‘alright be more careful right now’.” (03-98-004)*

#### 3.3.1 Understanding the body

Chronic fatigue is a long-term condition, and individuals experiencing it often need to make lifestyle adjustments. It is important for people suffering from chronic fatigue to accurately identify factors that could exacerbate their condition. The participants shared that the ability to identify these factors is an essential step in managing their condition. Some of the triggers identified by the participants included hot weather or sun exposure, skipping a nap, catching a cold, being in stressful situations, and adding more tasks to their usual routine.

*“… over the years, as I've aged, I've learned how to manage it. So, now when I get a little flu-a little funny, I say I need to rest. I need to go and ‘sit back’ and I will.” (01-98-001)*

Aside from knowing their own triggers, the participants also shared how they have developed unique ways of assessing their fatigue levels based on past experiences and patterns they have noted over time. For example, one participant measures the severity of fatigue based on how they feel the morning after a night of adequate sleep. Another participant used the need for a nap not only to measure fatigue but also to determine whether they are still within their exertional limits, “*When I feel the need to nap, I feel I have done too much” (07–98-002).* Although some individuals have identified specific patterns or signals that help assess their condition, there are also some who struggle with the ambiguity of fatigue as a symptom. One participant explained *“I put 9 for last week [in the activity log], but I thought of putting 10. I do not know what a 10 is. You know, it [rating fatigue] always has to be relative to something else” (01–98-002).*

#### 3.3.2 Discovering what works

The unique presentations and patterns of fatigue create a challenge in designing standardized algorithms. While there is abundant literature discussing the common features of chronic fatigue and how they can be managed, it is important for individuals to discover what works for them. Our participants have noted several interventions that they employ to manage their fatigue. The most common intervention our participants used was ensuring adequate sleep and daytime naps. *“I have built in rest time during my day; and I work really close to home so I can go back to my apartment in between my jobs and like lay down and basically close my eyes.”* Some participants benefited from utilizing paid services for some chores during the exacerbation of fatigue. It is important to recognize, however, that the use of paid services may be financially challenging for persons with limited economic resources.

It is also important to plan and appropriately pace activities. For example, a participant described being fatigued as *“it takes me a long time to start my day.”* As such, people experiencing chronic fatigue should be given extra time to prepare for certain tasks or activities. While they may benefit from having a structured routine where activities are anticipated and paced, it is also important that routines are kept flexible to accommodate the need for additional rest periods or alternative activities when necessary.

*“I start to think about how to do things as opposed to just doing things. So, [with] standing up, I say ‘What do I have to do to stand up?’ The best way I can describe this is it's like being a little kid who's just learning to walk.” (08-98-001)*

## 4 Discussion

The participants’ narratives revealed that fatigue is a unique personal experience. Another important finding of this study was how the participants coped with the multidimensional impact of chronic fatigue. Understanding how individuals experience this condition, how it impacts their lives, and how they cope are important aspects to consider in care planning.

Responses received from study participants highlight the importance of utilizing personal experiences in symptom management for chronic fatigue. In this context, personal experiences may include the individual’s understanding of their condition, established metrics for symptom management, interpersonal dynamics of their support system, and coping strategies. Table 2 presents examples of important points from the interviews that may be helpful in guiding clinicians when planning care for persons experiencing chronic fatigue. Based on the important points drawn from the experiences of the participants, clinicians may consider using more varied descriptors of chronic fatigue aside from “being tired” or “exhausted.” It is important to measure chronic fatigue based on descriptions that are familiar and meaningful to the person experiencing the symptom.

The descriptions of chronic fatigue experience shared by the participants, even those not diagnosed with ME/CFS, have significant similarities with the clinical diagnostic criteria of ME/CFS established by different groups in the past 20 years. For example, “unrefreshing sleep” has been in the clinical diagnostic criteria of the CDC (1994), the Canadian ME/CFS Case Criteria (2003), and the NAM’s (2015) criteria. The description of fatigue in relation to and the self-assessment of fatigue in relation to sleep have been described by our participants. However, assessment of energy levels in relation to sleep is not well captured in instruments to measure fatigue that are commonly used today such as the Fatigue Assessment Scale (FAS) and the FACIT-Fatigue. This underscores the potential need of including “unrefreshing sleep” as a descriptor of chronic fatigue in commonly used fatigue questionnaires. In addition, it is also important for clinicians to recognize how patients describe chronic fatigue using non-medical analogies. In this study, some participants compared chronic fatigue with “being ran over by a Mack truck,” starting the day with “less than 100% of battery” and feeling “like hitting a wall.” Other analogies identified by other studies include “feeling like zombie” and being a “wet fish” (30).

Finding a definitive way to measure chronic fatigue may be challenging since this symptom presents differently between people. A patient’s self-description of their chronic fatigue remains the best method to assess the impact of fatigue on someone’s life (31). The impact of chronic fatigue extends beyond the person experiencing it as it affects interpersonal interactions and the performance of social roles. For example, individuals with chronic fatigue may report a decline in social participation, work performance, and productivity (32, 33). In this study, participants discussed specific aspects of cognitive functions that they experience with chronic fatigue, such as difficulty following or understanding texts and difficulty finding the right words. However, currently available assessment tools such as the Fatigue Assessment Scale (FAS) only account for the frequency of experiencing mental exhaustion and difficulty in concentrating or thinking clearly but do not identify specific cognitive tasks (34). Including assessment of specific cognitive tasks such as understanding verbal information, performing basic calculations, and following directions may be beneficial in providing a better measure not only of cognitive limitations but also of how these limitations impact a person’s ability to perform activities of daily living (35).

The results of this study are similar to the findings of a meta-analysis that explored fatigue experiences of individuals with five non-infectious diseases (heart failure, multiple sclerosis, rheumatoid arthritis, chronic kidney disease, and chronic obstructive pulmonary disease) which included “feeling misunderstood” and “fear of being not believed” as among the themes that emerged when patients describe their fatigue experience (36). There may be a dissonance between how others perceive the impact of chronic fatigue on individuals experiencing the condition (36). Individuals who experience chronic fatigue may struggle to adjust to changing family dynamics or deal with a perceived loss of self and limited social network (37). The inclusion of topics regarding how to effectively communicate their condition and how to set healthy boundaries in the care plan would be beneficial. These skills may reduce the negative impact of chronic fatigue on their social wellbeing and lessen stressful interactions that may trigger the exacerbation of chronic fatigue.

Management of chronic fatigue should not only focus on momentary relief of symptoms, but empowering individuals with chronic fatigue to navigate the complexities of their condition should be equally considered. It is recommended that the assessment of chronic fatigue includes an assessment of the individual’s strengths, preferences, and available support system that could help them manage their symptoms. Strength-Based Nursing (SNB) model is an approach that can guide the plan of care for individuals with chronic fatigue. Unlike models of care that focus on disease and deficits, SBN underscores the importance of focusing on strengths within and outside of the person receiving care that can empower them to take control of their own health. The SBN model posits that health and wholeness can coexist with illness (38). In caring for individuals with long-term conditions, such as chronic fatigue, it is important to enhance their ability to adapt to the changes caused by their condition.

It is also important to note that the presentation and severity of chronic fatigue may vary or fluctuate over a period of time. The Ecological Momentary Assessment (EMA) aims to capture symptoms or behavior in the person’s everyday context. It recommends repeated collection of an individual’s current or very recent state, rather than one-time collection of a recall summary (39). Following the EMA model, clinicians and researchers can see how symptoms are experienced in an ecological context—meaning how it varies across different times and situations. When applied in the context of chronic fatigue, EMA would potentially improve the understanding of patterns, exacerbating factors, and environmental factors that affect people’s fatigue experience.

### 4.1 Limitations

It should be noted that the findings of this study are based on a small (*n* = 16), non-randomly selected sample. As such, the results of this study cannot be generalized and only apply to the sample population at the time of the interview. This study is based on participants’ subjective descriptions of their own chronic fatigue experiences, which were not correlated with any quantitative measures or biological markers of chronic fatigue. The pathophysiologic effect of diseases, the side effects of treatment procedures or medications, and the role of sociodemographic factors were not controlled in this study. Triangulation across methodologies and sources may be necessary to have a more comprehensive understanding of this phenomenon. It is also important to note that people’s interpretations of their experiences may vary across times and contexts. Understanding the potential influence of clinical and sociodemographic factors on fatigue experience is also vital.

## 5 Conclusion

Chronic fatigue is a unique personal experience, and descriptions of chronic fatigue are shared across medical diagnoses. Although the result of this study is not generalizable, it offers valuable information that can be used in designing quantitative measures for research and clinical use that are more reflective of patients’ experiences. Understanding a symptom from the perspective of the person experiencing it remains beneficial. Depending on the chronic fatigue experience and how the symptom affects the person’s ability to function, clinicians must constantly involve patients in finding ways to understand their symptom experience. Combining both objective measures and the patients’ subjective experience will help clinicians provide interventions that address the holistic needs of patients.

## Data availability statement

The datasets presented in this article are not readily available because the parent study is still ongoing. Requests to access the datasets should be directed to LS (saliganl@mail.nih.gov).

## Ethics statement

The studies involving humans were approved by the Institutional Review Board of the National Institutes of Health in Bethesda, Maryland, United States. The studies were conducted in accordance with the local legislation and institutional requirements. The participants provided their written informed consent to participate in this study.

## Author contributions

CR: Investigation, Methodology, Validation, Writing – original draft, Writing – review & editing, Formal analysis, Conceptualization. GG: Formal analysis, Validation, Writing – original draft, Writing – review & editing. CB: Data curation, Investigation, Project administration, Validation, Writing – review & editing. AR: Writing – review & editing, Validation, Project administration, Investigation, Data curation. LS: Conceptualization, Investigation, Methodology, Project administration, Resources, Supervision, Writing – review & editing.
